# Targeting Chronic Pain in Primary Care Settings Using Behavioral Health Consultants: A Pilot Study Implementing Brief Cognitive Behavioral Therapy for Chronic Pain

**DOI:** 10.1007/s10880-025-10121-0

**Published:** 2026-01-19

**Authors:** Jeffrey L. Goodie, Kathryn E. Kanzler, Cindy A. McGeary, Stacey Young-McCaughan, Alan L. Peterson, Briana A. Cobos, Anne C. Dobmeyer, Christopher L. Hunter, John Blue Star, Aditya Bhagwat, Timothy T. Houle, Jill C. Buhrer, Paul Fowler, Nicole Brackins, Melody R. Cardona, Donald D. McGeary

**Affiliations:** 1 Department of Family Medicine, Uniformed Services University of the Health Sciences, Bethesda, USA; 2 Department of Medicine, Baylor College of Medicine, Houston, USA; 3 Department of Psychiatry and Behavioral Sciences, The University of Texas Health Science Center at San Antonio, San Antonio, USA; 4 South Texas Veterans Health Care System, San Antonio, USA; 5 Medical Affairs, Defense Health Agency, Falls Church, USA; 6 C&C Hunter Consulting, Hawaii, USA; 7 Wilford Hall Ambulatory Surgical Center, San Antonio, USA; 8 Department of Anesthesia, Massachusetts General Hospital, Boston, USA; 9 Carl R. Darnall Army Medical Center, Fort Hood, USA

**Keywords:** Chronic pain, Primary care behavioral health, Military health system, DVPRS

## Abstract

Chronic pain is common among military service members, retirees, and families, yet non-pharmacologic options in primary care are limited. This pilot study evaluated the feasibility, and outcomes of Brief Cognitive Behavioral Therapy for Chronic Pain (BCBT-CP) delivered by integrated Behavioral Health Consultants (BHCs) in a Military Health System (MHS) clinic. This prospective, observational pragmatic pilot study recruited 44 Department of Defense beneficiaries (M age = 44.5 years [SD = 9.3 years]; 75% female; 50% Caucasian; 47.7% military family members, 38.6% retirees) with chronic pain (≥ 12 weeks). As part of usual care, participants received BCBT-CP, a modular skills-based intervention delivered within the Primary Care Behavioral Health model. Measures included the Defense and Veterans Pain Rating Scale (DVPRS), the Pain, Enjoyment of Life, and General Activity (PEG-3) scale, and the Behavioral Health Measure (BHM-20), assessed at baseline, during treatment, and 3- and 6-months after the baseline assessment. Feasibility was high, with 44 of 45 approached patients enrolling in the study. These participants attended a median of 3 appointments (IQR 2–6) over 52 days (IQR 22–110.5). Significant improvements were observed on DVPRS pain intensity (*p* < .001) and interference with activity, sleep, and stress. PEG total and subscales also improved (*p* < .05). No significant changes were detected on the BHM-20. Follow-up outcome data suggested partial loss of gains due to small sample size and attrition. Using BHCs to implement BCBT-CP in MHS primary care is feasible and improves short-term pain outcomes. Larger trials should test strategies to sustain benefits.

**Supplementary Information:**

The online version contains supplementary material available at 10.1007/s10880-025-10121-0.

Chronic pain conditions are a widespread problem among active duty members, retirees, and their family members. As many as 50% of deployed military members are expected to return with pain conditions that account for an estimated 73% of U.S. Army disability benefits paid each year (Pujol et al., 2015; Toblin et al., 2014). Direct and indirect costs due to chronic pain among military service members and veterans are high, and a 2005 report by the Department of Defense (DoD) Military Injury Prevention Priorities Working Group and the Army Medical Surveillance Activity found that orthopedic pain and injury account for over 24 million days of limited duty and a DoD-wide cost of $1.5 trillion due to direct and indirect costs associated with managing orthopedic conditions. A recent systematic review of chronic non-cancer pain in military veterans found a pooled chronic pain prevalence of 45% in a combined sample of over 14 million veterans (Qureshi et al., 2025) and a cross-sectional study of 29,802 U. S. adults from the National Health Interview Survey showed that military veterans were most likely to develop chronic pain associated with severe headache, facial pain, spinal pain (including neck), and joint pain (Taylor et al., 2024). Unfortunately, information about chronic pain among military dependents is sparse, though what has been published suggests that military dependents may be at greater risk for chronic pain than the family members of non-veterans (Noyek et al., 2023). Chronic pain is increasingly prevalent, and without the implementation of highly accessible, nonopioid, and effective treatment approaches, associated healthcare costs are likely to rise, placing additional burden on the DoD and Veterans Affairs (VA) healthcare systems (Office of the Army Surgeon General, 2010; Sherry et al., 2021).

Most patients with chronic pain are treated in primary care settings (Mills et al., 2016). Despite the significant needs of patients with chronic pain, the average length of time that a primary care physician spends discussing treatment of chronic pain with patients is 2.3 min (Tai-Seale et al., 2011). Treatment approaches in the military for chronic pain have traditionally focused on medical interventions, including the use of medications (e.g., opioids) and surgeries (McGeary et al., 2012, 2013). There is a significant need for research testing non-pharmacological pain management programs that can curtail the use of these interventions, especially studies focused on the implementation and utilization of these treatments. Non-pharmacological pain management interventions are underutilized in primary care compared to pharmacological treatments for numerous reasons, including provider and patient attitudes toward non-pharmacological treatments, skepticism about the efficacy of these treatments, lack of training and case consultation, and difficulty promoting these alternatives once opioid medications are prescribed (Becker et al., 2017; Roseen et al., 2024). Non-pharmacological pain management strategies are generally cheaper, more easily disseminated and used, and are safer than most medical interventions (Bushnell et al., 2021), so more evidence to support their use could have a significant impact on improving patient care in the long term. The most notable gaps in DoD pain management include the need for non-medication-based behavioral interventions for pain management in primary care (where the most patients with pain are being seen), consideration of military relevance among pain management programs to ensure their uptake among military service members with chronic pain, and the need for chronic pain management programs that can demonstrate decreased persistent opioid use for pain management, an intervention that has been shown to be harmful due to increased risk of opioid use disorder, overdose risk and death (Sandbrink et al., 2023).

The Defense Health Agency (DHA; i.e., the agency that manages operations within the military health system [MHS]) recognized the chronic pain problems highlighted in the Army Pain Management Task Force Report and the need for streamlined pain management services featuring greater availability of non-pharmacological pain management strategies in the wake of the growing opioid crisis. In June 2018, the DHA published the Procedural Instruction (DHA-PI) 6025.04, which required the establishment of a stepped-care model focused on non-pharmacologic pain treatments and minimizing the use of opioid medication for chronic pain (DHA, 2018). One important component of this stepped-care model was the use of behavioral health consultants (BHCs) integrated into primary care settings.

BHCs, who are typically psychologists or social workers, provide care in a manner that is consistent with the Primary Care Behavioral Health (PCBH) model of integration (Hunter et al., 2024; Robinson & Reiter, 2025). The PCBH model uses a population health focus to meet the behavioral health needs of those seen in primary care (Reiter et al., 2018). The BHC assists primary care clinicians (PCCs) with a full range of behavioral health concerns (e.g., anxiety, chronic pain, depression, insomnia, tobacco cessation) and typically sees patients for one to four appointments each 20 to 30 min in length (i.e., modeling the typical primary care appointment timeframe). The BHC may see patients more frequently for chronic concerns if those additional appointments help the primary care team manage the patient’s concerns. Often, patients experiencing chronic pain hesitate to follow-up on referrals to mental health clinics for similar care due to perceived stigma and feelings of rejection from medical care (Ojala et al., 2015). Because BHCs are embedded in primary care clinics, they can see patients sooner, free up primary care and specialty mental health resources, and implement non-pharmacological, behavioral health pain management strategies in a medical environment that minimizes perceptions of stigma and rejection. The PCBH model has widespread availability throughout the DoD. In August 2013, the DoD issued a policy requiring all the military services to place at least one BHC into each primary care clinic that had 3000 or more patient enrollees (i.e., roughly 296 clinics; (Hunter et al., 2014). DHA-PI 6025.04 required that BHCs throughout the DoD be trained in approaches for helping those experiencing chronic pain.

Evidence-based non-pharmacologic treatments for chronic pain have been adapted for implementation by BHCs. Beehler et al. (2017) developed a manualized, brief cognitive-behavioral therapy for chronic pain (BCBT-CP) for implementation throughout the Veterans Health Administration (VHA). BCBT-CP is based on cognitive-behavioral therapy for chronic pain (CBT-CP), a manualized non-pharmacological intervention for chronic pain that has been tested and implemented in specialty care settings within the VHA (cf. (Stewart et al., 2015)). BCBT-CP includes six appointments, each designed to last 30 min, and is a modular intervention to enhance self-management of chronic pain through education, skills training, and behavior change strategies. The program begins with pain education and goal setting, followed by modules that address activity pacing, relaxation training, and cognitive coping skills to identify and modify unhelpful thoughts related to pain. The final appointment focuses on developing a personalized pain action plan to support maintenance of gains and continued use of skills after treatment completion (Beehler et al., 2017, 2019). Beehler et al. (2019) found that among veterans (i.e., mean age 51.4 years; 75% male) receiving the BCBT-CP intervention in a VHA facility, ratings on the Pain, Enjoyment, General Activity (PEG) scale improved over the first 3 appointments, with minimal change after those visits. These participants completed a mean of 4.9 appointments, with 47% completing all 6 appointments. Among 34 participants who completed a post-treatment survey, most participants indicated that they received “just the right” number of appointments and that the length of the appointments was “just right” (Beehler et al., 2021). While Beehler et al. and’s (2019, 2021) support the use of BCBT-CP within the VHA, these studies relied on limited post-treatment follow-up and quality improvement designs that did not evaluate sustained clinical outcomes or broader implementation under routine care conditions. Further research is needed to evaluate the implementation and longitudinal impact of BCBT-CP in more diverse, real-world settings, particularly within DoD primary care clinics, where BHCs serve an often younger, more diverse, and operationally distinct population of active duty service members, retirees, and family beneficiaries. A pragmatic approach is essential to assess the feasibility, effectiveness, and potential scalability of BCBT-CP across the broader MHS.

VHA and DHA personnel, including co-authors on this manuscript (CH, AD), revised the brief version (BCBT-CP) for implementation by BHCs throughout the MHS (Beehler et al., 2018). These adaptations ensured that the manual was consistent with how BCBT-CP is delivered within the MHS—for example, incorporating an assessment into Module 1 followed by a brief intervention suitable for the first visit; structuring the first module around the 5A’s approach; including required DHA assessment measures and procedures; allowing that not all patients will require all modules, and integrating case examples relevant to military populations (i.e., active duty service members and their families). Multi-disciplinary providers assigned to primary care clinics throughout the MHS were trained on the DHA Stepped Care Pain Pathway. The BHCs in these clinics were trained to use the BCBT-CP using an 8-h video-teleconference or in-person training followed by consultation telephone calls with DHA BCBT-CP trainers. Training was supplemented using an online chronic pain subject matter video that was developed by one of the study PIs (DM).

The purpose of this pilot study was to (1) evaluate the feasibility (i.e., recruitment and retention) of implementing the BCBT-CP in a DHA primary care clinic and to (2) explore preliminary trends in functioning of service members and family members experiencing chronic pain following participation in the intervention, and whether any observed changes were sustained over time. Because there was no control group, analyses of change and maintenance were exploratory and intended to inform the design and focus of future controlled trials rather than to draw causal conclusions about intervention efficacy.

## Method

This pilot study used a prospective, observational pragmatic design. The study design and procedures were developed with the goal of maintaining a pragmatic research approach as evaluated using the PRECIS-2 tool (Loudon et al., 2015). Pragmatic designs are intended to assess the effectiveness of interventions in real-world clinical settings (Ford & Norrie, 2016). The PRECIS-2 tool provides a structured method to rate how closely a study’s design aligns with this goal across domains such as eligibility, setting, and delivery flexibility (Loudon et al., 2015; Thorpe et al., 2009). Results of our PRECIS-2 tool evaluation and pragmatic design considerations are described in Kanzler et al. (2022).

This study was approved by the University of Texas Health Science Center at San Antonio's Institutional Review Board acting as the single IRB with the Carl R. Darnall Army Medical Center and the Uniformed Services University relying upon the regulatory review under a Defense Health Agency General Reliance Agreement. The U. S. Army Medical Research and Development Command Office of Human Research Protection monitored the regulatory approvals in accordance with the funded award.

### Participants

Participants were recruited from a primary care clinic affiliated with Carl R. Darnall Army Medical Center at Fort Hood, TX, and serving active duty servicemembers, retirees and their family members. Consistent with pragmatic trial designs, we minimized exclusion criteria (Kanzler et al., 2022; Louden, 2015). Eligible participants included (a) DoD beneficiaries 18 years and older, (b) with a PCC working in the clinic, (c) referred for pain management to the BHC, and (d) had at least one pain condition for 12 weeks or more in duration (as diagnosed by the PCC and consistent with the most recent definition of chronic pain by the International Association for the Study of Pain; Treede et al., 2019). Exclusion criteria for patient participants included (a) those who presented with symptoms of psychosis and/or delirium based on clinical judgment and medical record review, (b) a medical condition or life circumstance that would contraindicate or prevent participation (e.g., scheduled surgery), and (c) an inability to comprehend the informed consent process or study instructions.

### Procedure

Patient participants were recruited from the clinical population seen by PCCs and referred to BHCs at the participating clinic for chronic pain management. There were no changes made to typical clinic functioning, except to increase reminders to clinicians and clinic staff that BHCs are an important resource for treating chronic pain. Study staff reviewed the medical records of patients being seen in the clinic for chronic pain and alerted the PCCs and the BHC of patients who might be potential participants. Prospective study patient participants were referred to the research coordinator by the BHC for consent either as a direct hand-off from the BHC (i.e., the study staff met the potential participant immediately after the BHC appointment) or through a consent to contact form the participant completed when seeing the BHC (i.e., research staff contacted the potential participant at a date after the initial BHC appointment and prior to the second appointment with the BHC). Patients who did not agree to participate in the study allowing the study team to track their outcomes or who did not agree to be contacted could still receive BCBT-CP to address their chronic pain, as this was the established standard of care for BHCs. Screening of patient participants for inclusion and exclusion criteria was performed during the baseline assessment.

### Interventions

As summarized in Table 1, the BCBT-CP used in this study included seven modules: Module A: Assessment, engagement, and goal setting; Module B: Pain Education and Relaxation Training; Module C: Activities and Pacing; Module D: Relaxation Training; Module E: Cognitive Coping 1 (identifying pain thoughts); Module F: Cognitive Coping 2 (changing pain thoughts); Module G: Pain Action Plan. DHA guides BHCs to complete Modules A, B, G and at least one additional module to consider treatment “complete.” The BHC managed the consented study participants as they would any patient not participating in the study.

**Table 1:** Brief cognitive behavioral therapy for chronic pain (BCBT-CP) modules

Module	Content	Description
A	Assessment, Engagement, and Goal Setting	Focused biopsychosocial assessment of pain, describing and engaging in BCBT-CP
B	Education and Relaxation Training 1	Education on chronic pain, overview of relaxation and instruction in diaphragmatic breathing
C	Activities and Pacing	Importance of engagement in activities using a planned approach
D	Relaxation Training 2	Progressive muscle relaxation and guided imagery
E	Cognitive Coping 1	Recognize unhelpful thoughts that negatively impact the pain experience
F	Cognitive Coping 2	Modify thoughts that are unhelpful when managing pain
G	The Pain Action Plan	Plan for independent implementation of BCBT-CP skills and identify follow-up needs

Behavioral Health Consultants (BHCs) were expected to provide module A, B, G, and at least one other module for the intervention to be considered complete

The BHC was expected to administer the Behavioral Health Measure (BHM-20) and Defense and Veterans Pain Rating Scale (DVPRS) at each visit as a standard of care procedure. During the first appointment, the BHC conducted an initial assessment and provided brief education about chronic pain consistent with Module A; however, no BCBT-CP intervention content was delivered at that time. Research staff contacted potential participants after their first appointment and collected demographic and military service information, administered study measures, including readministering the BHM-20 and DVPRS for research purposes. Participants were contacted 3 months and 6 months after their first appointment with the BHC at which time study measures were again administered.

### Post-Treatment

Because this study used a pragmatic design, operationally defining the treatment endpoint was complicated. In routine primary care, the conclusion of BHC services is not standardized and may occur either through a mutually agreed-upon termination of care or when a patient discontinues attendance, both of which represent typical patterns of PCBH service delivery. Accordingly, we defined the end of care as the date of the last documented BHC encounter. Any assessments completed after that date were classified as post-treatment and used to evaluate outcomes following the cessation of active clinical contact with the BHC.

### Measures

To assess change in both pain-related outcomes and overall mental health functioning, participants completed standardized, validated self-report measures at baseline, during treatment, and at follow-up. Per standard of care, BHCs were expected to administer these instruments at every appointment.

#### Feasibility

Feasibility was defined in terms of recruitment and retention of participants throughout the study period. Recruitment feasibility reflected the proportion of eligible patients who agreed to participate when referred by the BHC to participate. Retention feasibility was defined by the extent to which participants remained engaged with the intervention and completed follow-up assessments. These indicators were selected to capture the practicality of identifying, enrolling, and maintaining patient participation within the study and the care offered by the BHC.

#### Defense and Veterans Pain Rating Scale (DVPRS) and DVPRS Supplemental Questions

The DVPRS (Buckenmaier et al., 2013) is a standardized pain rating scale combining features of numeric and graphic rating scales of pain that measures current pain. The DVPRS and DVPRS Supplemental Questions include 5 items, each rated on a 0–10 numeric rating scale assessing current pain and pain interference on general functioning, impact of pain on sleep, and impact of pain on mood. DVPRS assessment is tailored specifically to military and veteran pain sufferers and has shown good reliability and validity in these populations (Buckenmaier et al., 2013; Polomano et al., 2016). For this pilot study, we included an additional item on the DVPRS to assess pain intensity over the past week, in addition to the item that asked about current pain. Consistent with prior work and the intended multidimensional use of the DVPRS (Buckenmaier et al., 2013; Polomano et al., 2016), items were analyzed individually rather than combined into a total score to capture distinct aspects of pain intensity and interference across functional domains.

#### Behavioral Health Measure (BHM-20)

The BHM-20 is a 20-item questionnaire that assesses both intensity and frequency of mental health distress and functioning based on the following scales: Well-Being (3 items), Psychological Symptoms (13 items), and Life Functioning (4 items) measured through a Likert rating scale (Kopta & Lowry, 2002). A Global Mental Health scale is derived by summing all 20 items and provides the most meaningful index of functioning due to high inter-correlations between subscales. The BHM-20 discriminated effectively between clinic and non-clinic samples, and the BHM-20 scales showed mostly high correlations with similar scales of four other well-established mental health questionnaires (Kopta & Lowry, 2002). The BHM-20 has been validated for use by BHCs with military beneficiary primary care patients (Bryan et al., 2014).

#### Pain, Enjoyment of Life, and General Activity (PEG-3)

The PEG-3 is a 3-item assessment of pain developed as a valid, reliable, and “ultra brief” measure of pain, derived from the items and structure of the Brief Pain Inventory (Krebs et al., 2009). The PEG-3 includes three items scaled from 0 to 10 for which individuals rate pain intensity on average over the past week, interference of pain on enjoyment of life, and interference of pain on general activity. PEG-3 has been used in numerous pain trials since its development including studies of the CBT-CP intervention upon which BCBT-CP is based (cf. Heapy et al., 2020). Reliability and validity are well-established, and the minimal clinically significance difference has been estimated at 1 to 2 points (Reed et al., 2024).

### Statistical Analysis

To examine changes in the DVPRS, BHM-20, and PEG-3 over the course of treatment, we utilized linear mixed-effects models, with random intercepts at the level of the individual, that accommodated the varying number of appointments for each participant. The pilot study was conducted, in part, to determine the effect size of the BHC intervention on common measures of improved functional outcomes. Given the average standard deviation (.66, range .60–.75), and the average within-subject correlation (.78, range .71–.83) across BHM-20 subscales (Kopta et al., 2015), and a Type 1 error rate of .05 (2 tailed), 38 participants were needed to find a difference in mental health using the BHM-20 with an effect size of .20 with a power of .80.

## Results

A total of 44 participants were recruited for the study between June 2019 through January 2020. Table 2 summarizes the demographics of the sample. The mean age of participants was 44.5 years (SD = 9.3 years). Most participants were female (75%). Fifty percent of participants identified their race as White/Caucasian, 34% as African American, and 16% as other. Most participants were either military family members (47.7%) or retirees (38.6%) and were affiliated with the Army (93.1%).

**Table 2:** Sample demographic characteristics (N = 44)

Demographic	Category	N (%) or Mean ± SD
Sex	Male	11 (25.0)
	Female	33 (75.0)
Age		44.5 ± 9.3
Ethnicity	Hispanic	7 (15.9)
	Non-Hispanic	37 (84.1)
Race	African American	15 (34.1)
	Caucasian	22 (50.0)
	Other	7 (15.9)
Marital Status	Currently married/Living with partner	39 (88.7)
	Currently separated or divorced	5 (11.4)
Military Status	Retired	17 (38.6)
	Veteran	6 (13.6)
	Family Member	21 (47.7)
Branch	Army	21 ( 91.3)
	Navy	1 ( 4.3)
	Air Force	1 ( 4.3)
Employment Status	Full Time	19 (47.5)
	Part Time	7 (17.5)
	No Regular Employment	2 ( 5.0)
	Unemployment	12 (30.0)
Household Income	10 to 35 K	3 ( 7.7)
	35 to 50 K	7 (17.9)
	50 to 100 K	22 (56.4)
	> 100 K	7 (17.9)
BHC Appointments		3.73 ± 1.71

### Feasibility

**Fig. 1:**
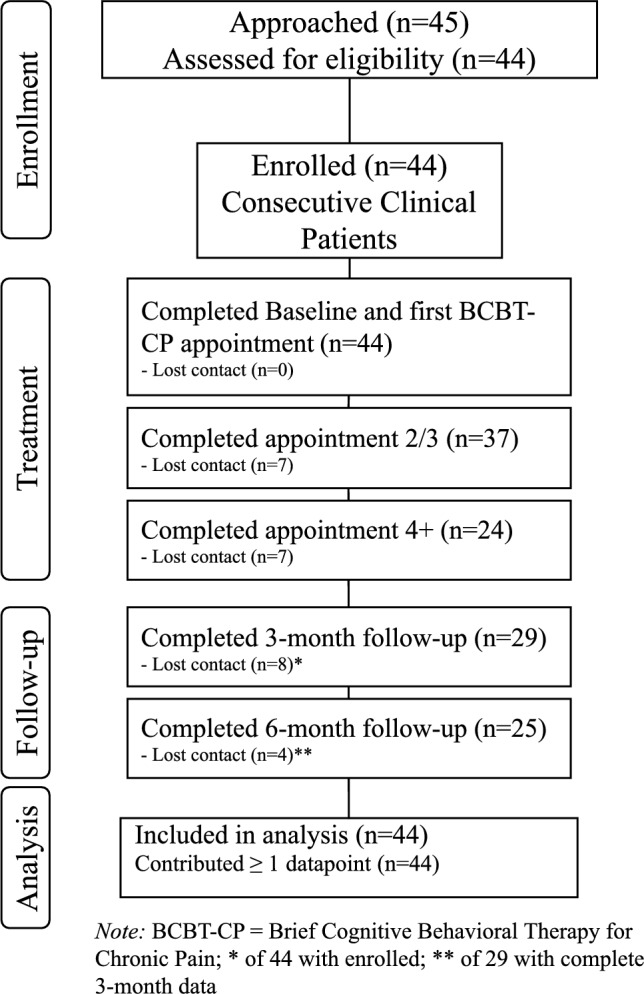
CONSORT chart for study enrollment

Forty-four individuals were observed for a median [25th, 75th] of 3 [2, 6] appointments that occurred over 52 [22, 110.5] days. Among 45 individuals approached about participating, 44 enrolled in the study suggesting that implementation of BCBT-CP in primary care was deemed desirable by MHS beneficiaries experiencing chronic pain. In this sample, 54% (n = 24) of participants attended four or more appointments, and a small subset (9%; n = 4) completed seven appointments. To assess differences between treatment completers and non-completers (n = 8), standardized mean differences (SMDs) were calculated for key demographic variables and baseline pain rating on the DVPRS using the R ‘tableone’ package. As shown in Supplemental Table 1, there were large imbalances between the completer groups based on race, marital status, and education, with moderate imbalances for current and average pain. A CONSORT chart for study enrollment is provided in Fig. 1.

### Changes in Pain and Mental Health Functioning

#### Pain, as Assessed with the DVPRS

At baseline, current pain was rated as a 4.7 [4.0, 5.3] (based on a standalone pain intensity item). Effect estimates were developed for each of the 6 DVPRS items and scaled to represent change over a median 52-day treatment period (based on our findings that most participants in the pilot trial engaged in BCBT-CP treatment with their BHC). Item effect estimates are summarized in Table 3. In short, all but one DVPRS item showed statistically significant improvement over time. Participants reported improvement in current pain ratings of -0.5 points [-1.0, -0.1; *p* = .029] on the DVPRS, while improvement over the past week was -0.9 points [-1.2, -0.5; *p* < .001]. Notably, the decrease in pain over the past week exceeded the minimum clinically significant difference for the DVPRS of -0.75 (cf. Boyer et al., 2022), suggesting that pain improvement over the past week was both statistically and clinically significant. DVPRS supplemental items revealed significant improvements over the median 52-day BCBT treatment period of -1.2 points [-1.7, -0.6; *p* < .001] for interference of pain on activity, -1.1 points [-1.7, -0.4; *p* = .002] for interference of pain on sleep, and -0.7 points [-1.3, -0.1; *p* = .026] for interference of pain on stress.

**Table 3:** Change in defense and veterans pain rating scale (DVPRS) and pain, enjoyment of life, and general activity (PEG-3) (N = 25 to 44)

DVPRS Question	Intercept	95%CI	Slope	95%CI	p
Current Pain	4.7	4.0, 5.3	− 0.5	− 1.0, − 0.1	.029
Average Pain Past Week	5.6	5.1, 6.2	− 0.9	− 1.2, − 0.5	< .001
Usual Activity Interference	5.4	4.7, 6.1	− 1.2	− 1.7, − 0.6	< .001
Sleep Interference	5.4	4.6, 6.2	− 1.1	− 1.7, − 0.4	.002
Affected Mood	4.5	3.8, 5.3	− 0.5	− 1.1, − 0.1	.125
Impacted Stress	4.6	3.8, 5.4	− 0.7	− 1.3, − 0.1	.026

PEG-3 Question					
Average Pain (past week)	5.9	5.3, 6.5	− 1.3	− 2.0, − 0.6	< .001
Enjoyment Interference	5.8	5.0, 6.6	− 1.1	− 2.0, − 0.2	< .015
Activity Interference	5.9	5.1, 6.6	− 1.2	− 2.1, − 0.4	.007
Total Score	5.9	5.2, 6.5	− 0.7	− 1.2, − 0.3	.003

#### Mental Health Functioning as Assessed with the BHM-20

On the BHM-20, as summarized in Table 4, we did not observe significant changes in reported functioning during treatment or post-treatment (*p* > .05).

#### Pain as Assessed with the PEG-3

Observations for change on the PEG-3 were like those for the DVPRS. Participants reported a statistically significant mean change in the total PEG-3 score across a median 52-day treatment period of -0.7 points [-1.2, -0.3; *p* = .003], pain intensity by a mean -1.3 points [-2.0, -0.6; *p* < .001], interference of pain on enjoyment of life of a mean -1.1 points [-2.0, -0.2; *p* = .015], and interference of pain on general activity of a mean -1.2 points [-2.1, -0.4; *p* = .007]. The PEG-3 total score change of -0.7 was below the threshold for minimal clinically important difference (bounded between -1.0 and -2.0; cf. Reed et al., 2024) but all three item scores were within the range of a clinically significant difference.

#### Post-Treatment

**Fig. 2:**
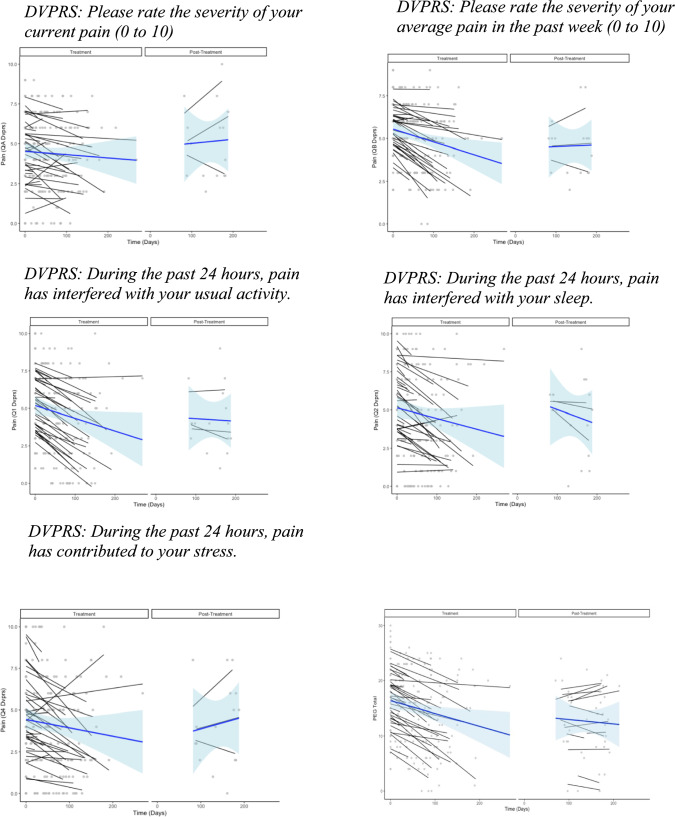
Individual responses to Defense and Veterans Pain Rating Scale (DVPRS) questions and the Pain, Enjoyment of Life, and General Activity (PEG) during and following treatment. The overall effect is summarized by a blue line with blue shading representing error around the summary line. Each gray dot represents a treatment session or post-treatment assessment for which a score was given

Assessments that were conducted post-treatment (i.e., after the last BHC appointment and 3- and 6-months after the baseline assessment) were visualized using spaghetti plots due to significant dropout in follow-up assessments and the relatively small sample size for this pilot project (see Fig. 2). Plots show individual trajectories of treatment response based on DVPRS item scores and the PEG-3 total score during the treatment and post-treatment study phases with blue trend lines summarizing the overall effects in each phase. Because this was a pragmatic trial, and due to the nature of the flexible/modular BCBT-CP intervention, treatment duration varied between participants based on their adherence to the clinical treatment regimen and the collaborative goals set with their BHC. Thus, trajectory lines in the spaghetti plot varied in length (i.e., treatment duration) across the participants; treatment duration is represented in days on the x-axis with time 0 representing the first BHC visit. Statistical changes during the treatment phase are summarized above. Post-treatment sample sizes were too small for inferential analysis, so examination of long-term outcomes was qualitative and should be interpreted with appropriate caution. Consistent with the above DVPRS and PEG-3 analyses, examination of individual trajectories showed some participants who were non-responders (participants with no significant change in scores) and some who significantly worsened during treatment, suggesting some heterogeneity of treatment effects in this population. Based on the scant data available, post-treatment outcomes of both measures revealed recidivism or worsening of pain symptoms during the post-treatment phase. The intercept value of the post-treatment phase (for those who completed post-treatment assessments) appeared to be similar to or worse than the intercept for pain scores at baseline. Though some participants reported improvement in pain from 3- to 6-month follow-up, most of those who supplied post-treatment data showed a worsening in post-treatment pain symptoms.

## Discussion

Overall, individuals engaged with the BHCs and attended follow-up appointments at a rate suggesting that the implementation of the intervention was feasible within this one DHA primary care setting. Recruitment feasibility was demonstrated by the high enrollment rate—44 of 45 approached patients—which indicated strong initial interest and willingness to participate. However, because feasibility was not measured using validated scales and attendance averaged three appointments over a median of 52 days, these findings should be interpreted cautiously. Thus, while our findings support the logistical feasibility of embedding chronic pain management within primary care, further research is needed to more rigorously assess patient acceptability, satisfaction, and barriers to sustained engagement. These findings reinforce prior research indicating that embedding behavioral health services in primary care facilitates uptake of psychosocial pain management strategies, particularly in military and veteran populations (Beehler et al., 2019; Hunter et al., 2014).

Examination of differences between treatment completers and non-completers further revealed that pilot participants who identified as Caucasian, were married, reported more education and higher baseline pain intensity were more likely to dropout of BCBT-CP treatment. Given the small sample size, these findings should be considered cautiously but suggest the possibility of systematic differences in treatment engagement based on demographics and pain that could affect future trials and implementation.

The number of appointments completed varied, reflecting the flexible and patient-centered nature of BCBT-CP. Rather than adhering to a prescribed number of visits, treatment duration is collaboratively determined by the patient and provider based on individual goals and progress. In this sample, over half of participants (54%) attended four or more appointments, while a smaller proportion (9%) completed seven. This variability aligns with the intended design of BCBT-CP, which emphasizes adaptability to patient needs and real-world feasibility within the constraints of primary care delivery.

**Table 4:** Change in Behavioral Health Measure (BHM-20)

Subscale	Intercept (SE)	Treatment Slope (95%CI) *p*	Post-treatment Slope (95%CI) *p*
Global Mental Health	3.0 (0.1)	− 0.0 (− 0.1 to 0.0) *p* = .480	0.0 (− 0.2 to 0.2) *p* = .939
Well-Being	2.2 (0.2)	0.0 (− 0.1 to 0.1) *p* = .748	0.0 (− 0.2 to 0.3) *p* = .863
Psychological Symptoms	3.3 (0.1)	− 0.0 (0 to 0) *p* = .162	− 0.0 (− 0.2 to 0.1) *p* = .804
Life Functioning	2.4 (0.1)	0.0 (− 0.1 to 0.1) *p* = .814	0.1 (− 0.2 to 0.4) *p* = .596

The most notable and clinically meaningful results emerged from the DVPRS and PEG-3, which both showed reductions in average pain intensity and pain-related interference with usual/general activity, life enjoyment, sleep, and stress during the course of treatment. These improvements are particularly important given the brief, population-health-oriented nature of BHC encounters in the PCBH model. Even within this short time frame and limited number of visits, patients demonstrated changes that are likely to translate into better day-to-day functioning and quality of life. The modular structure of BCBT-CP, allowing flexibility to tailor content to individual needs while maintaining a core set of skills, may have contributed to these observed benefits.

In contrast, the BHM-20 did not reflect statistically significant improvements in global mental health functioning. This discrepancy may reflect limitations of the BHM-20 in detecting condition-specific functional changes, as it is designed as a broad mental health measure rather than a targeted pain outcome. Given that DVPRS improvements were observed, it is possible that participants were experiencing meaningful changes in pain-related functioning that were not captured by the broader mental health domains measured by the BHM-20. This highlights the importance of selecting outcome measures that align closely with intervention targets, especially in pragmatic trials where small but clinically relevant gains can be overlooked if only global measures are used.

Follow-up data at 3- and 6-months suggest that some of the pain-related gains may diminish over time, with scores trending toward baseline levels. Although these findings must be interpreted cautiously due to the small number of participants available at follow-up and the potential for selection bias, they point to the potential value of booster contacts or ongoing low-intensity support to maintain treatment gains. This pattern is consistent with other chronic pain interventions, where ongoing skill reinforcement is often necessary to sustain benefits (Beehler et al., 2021).

It is unclear whether the lack of observed change reported on the BHM-20 was a function of the lack of change in functioning among participants or the inadequacy of the measure to detect changes that were occurring. Based on the responses observed on the DVPRS, it appears that participants were experiencing some changes. Power analyses implied that there were sufficient number of individuals recruited for the study if we had observed effect sizes similar to those observed in other studies.

Engagement and follow-up patterns in this study are consistent with prior evaluations of BCBT-CP delivered in PCBH settings. In a large VHA demonstration project, patients completed an average of approximately five appointments, with most attending all of the six appointments, and demonstrated significant improvements in pain intensity and functional interference by the third visit (Cohen’s *d* = 0.65; Beehler et al., 2019). Similarly, Beehler et al. (2021) found that 89% of patients reported high satisfaction and perceived improvement in pain-related functioning following BCBT-CP. Although participants in the present study completed a median of three appointments over a median of 52 days, comparable to the early response trajectory observed in prior work, these results further support the feasibility of brief, embedded pain interventions in primary care. Extending beyond veteran populations previously studied by Beehler et al., (2019, 2021), our pilot highlights the potential for meaningful functional gains within the limited session structure typical of PCBH practice. Future research should more systematically assess changes in mental health functioning (e.g., mood, stress) alongside pain outcomes to clarify behavioral mechanisms of improvement and sustainment.

Demographically, this sample differed from prior VA-based BCBT-CP studies (Beehler et al., 2019, 2021), with participants being predominantly female, younger, and more likely to be military family members. These characteristics may influence both the delivery and the sustainability of treatment effects. Family members of military service members and veterans may face unique role demands and care responsibilities that could impact their ability to consistently apply skills over time, suggesting an opportunity for tailoring BCBT-CP content to these specific contexts.

### Limitations

Several limitations warrant consideration. First, this was designed as a pilot study, and the small sample size and lack of a control group preclude causal inferences. Second, attrition at follow-up introduces the possibility of bias, as those who remained in the study may differ systematically from those lost to follow-up. Third, reliance on self-report measures may be affected by recall bias or social desirability. Fourth, although the BHC was trained how to deliver BCBT-CP, we do not know whether the BHC maintained fidelity to the trainings. Fifth, participants were not prohibited from seeking additional pain services while enrolled in this study, and by the nature of an integrated care setting, they may have been appropriately encouraged to engage in other treatments in their BHC visits. There is a possibility that any additional services received influenced study outcomes. Finally, while pragmatic in design, the study was conducted at a single military treatment facility with a single BHC, potentially limiting generalizability to other settings, BHCs, or service branches.

### Strengths

Despite these limitations, the study has notable strengths. It tested a manualized, evidence-based behavioral pain intervention in the real-world context of MHS primary care, maintaining high external validity. The trial achieved exceptional enrollment rates, reflecting patient receptivity to integrated behavioral care for pain. In addition, the use of both condition-specific (DVPRS) and global (BHM-20) measures provided a nuanced understanding of potential treatment effects.

## Conclusions

These pilot study findings support the feasibility of embedding brief, targeted cognitive-behavioral pain management interventions into primary care via the PCBH model. While short-term improvements in pain intensity and interference are encouraging, future research should examine strategies to sustain these benefits over time. This might include structured booster contacts, integration of digital or telehealth-based reinforcement tools, or stepped-care protocols that facilitate rapid re-engagement when symptoms worsen. Larger, multisite pragmatic trials with longer follow-up periods are warranted to determine the full impact of BCBT-CP on both pain-specific and global functioning outcomes in diverse military and family member populations.

## Supplementary Information

Below is the link to the electronic supplementary material.

Supplementary file1 (DOCX 16 KB)

## Data Availability

No datasets were generated or analysed during the current study.

## References

[CR1] Beehler, G. P., Dobmeyer, A. C., Hunter, C. L., Funderburk, J. S., S. (2018). *Brief cognitive behavioral therapy for chronic pain: BHC manual*. Defense Health Agency.

[CR2] Beehler, G. P., Loughran, T. A., King, P. R., Dollar, K. M., Murphy, J. L., Kearney, L. K., & Goldstein, W. R. (2021). Patients’ perspectives of brief cognitive behavioral therapy for chronic pain: Treatment satisfaction, perceived utility, and global assessment of change. *Families, Systems, & Health,**39*, 351–357. 10.1037/fsh0000606 10.1037/fsh0000606 34410777

[CR3] Beehler, G. P., Murphy, J. L., King, P. R., & Dollar, K. M. (2017). *Brief cognitive behavioral therapy for chronic pain: Therapist manual*. Department of Veterans Affairs.

[CR4] Beehler, G. P., Murphy, J. L., King, P. R., Dollar, K. M., Kearney, L. K., Haslam, A., Wade, M., & Goldstein, W. R. (2019). Brief cognitive behavioral therapy for chronic pain: Results from a clinical demonstration project in Primary Care Behavioral Health. *Clinical Journal of Pain,**35*, 809–817. 10.1097/AJP.0000000000000747 31318726 10.1097/AJP.0000000000000747

[CR5] Boyer, C. W., Lee, I. E., & Tenan, M. S. (2022). All MICDs are wrong, but some may be useful. *Journal of Orthopaedic & Sports Physical Therapy,**52*, 401–407. 35647882 10.2519/jospt.2022.11193

[CR6] Bryan, C. J., Blount, T., Kanzler, K. A., Morrow, C. E., Corso, K. A., Corso, M. A., & Ray-Sannerud, B. (2014). Reliability and normative data for the Behavioral Health Measure (BHM) in primary care behavioral health settings. *Families, Systems, & Health,**32*, 89–100. 10.1037/fsh0000014 24447151

[CR7] Buckenmaier, C. C., Galloway, K. T., Polomano, R. C., McDuffie, M., Kwon, N., & Gallagher, R. M. (2013). Preliminary validation of the Defense and Veterans Pain Rating Scale (DVPRS) in a military population. *Pain Medicine,**14*, 110–123. 10.1111/j.1526-4637.2012.01516.x 23137169 10.1111/j.1526-4637.2012.01516.x

[CR31] Bushnell, M. C., Frangos, E., & Madian, N. (2021). Non-pharmacological treatment of pain: Grand challenge and future opportunities. *Frontiers in Pain Research (Lausanne, Switzerland)*, *2*, 696783. 10.3389/fpain.2021.696783 10.3389/fpain.2021.696783 PMC8915661 35295445

[CR8] Defense Health Agency (DHA). (2018). Pain management and opioid safety in the military health system (MHS) 6025.04.

[CR34] Ford, I., & Norrie, J. (2016). Pragmatic trials. *The New England Journal of Medicine*, *375*(5), 454–463.10.1056/NEJMra1510059 10.1056/NEJMra1510059 27518663

[CR9] Heapy, A. A., Driscoll, M. A., Buta, E., LaChappelle, K. M., Edmond, S., Krein, S. L., Piette, J. D., Mattocks, K., Murphy, J. L., DeBar, L., MacLean, R. R., Ankawi, B., Kawecki, T., Martino, S., Wagner, T., & Higgins, D. M. (2020). Co-operative pain education and self-management (COPES) expanding treatment for real-world access (ExTRA): Pragmatic trial protocol. *Pain Medicine,**21*, S21–S28. 33313733 10.1093/pm/pnaa365 PMC7734659

[CR10] Hunter, C. L., Goodie, J. L., Dobmeyer, A. C., & Dorrance, K. A. (2014). Tipping points in the Department of Defense’s experience with psychologists in primary care. *American Psychologist,**69*, 388–398. 10.1037/a0035806 24820688 10.1037/a0035806

[CR11] Kanzler, K. E., McGeary, D. D., McGeary, C., Blankenship, A. E., Young-McCaughan, S., Peterson, A. L., Buhrer, J. C., Cobos, B. A., Dobmeyer, A. C., Hunter, C. L., Bhagwat, A., Star, J. A. B., & Goodie, J. L. (2022). Conducting a pragmatic trial in Integrated Primary Care: Key decision points and considerations. *Journal of Clinical Psychololgy in Medical Settings,**29*, 185–194. 10.1007/s10880-021-09790-4 10.1007/s10880-021-09790-4 PMC8184053 34100153

[CR12] Kopta, M., Owen, J., & Budge, S. (2015). Measuring psychotherapy outcomes with the Behavioral Health Measure-20: Efficient and comprehensive. *Psychotherapy,**52*, 442–448. 10.1037/pst0000035 26641374 10.1037/pst0000035

[CR13] Krebs, E. E., Lorenz, K. A., Bair, M. J., Damush, T. D., Wu, J., Sutherland, J. M., Asch, S. M., & Kroenke, K. (2009). Development and initial validation of the PEG, a three-item scale assessing pain intensity and interference. *Journal of General Internal Medicine,**24*, 733–738. 19418100 10.1007/s11606-009-0981-1 PMC2686775

[CR14] Loudon, K., Treweek, S., Sullivan, F., Donnan, P., Thorpe, K. E., & Zwarenstein, M. (2015). The PRECIS-2 tool: Designing trials that are fit for purpose. *BMJ,**350*, Article h2147. 10.1136/bmj.h2147 25956159 10.1136/bmj.h2147

[CR15] McGeary, D. D., Peterson, A. L., Seech, T., McGeary, C. A., Gatchel, R. J., & Vriend, C. (2013). Health care utilization after interdisciplinary chronic pain treatment: Part II. Preliminary examination of mediating and moderating factors in the use of costly health care procedures. *Journal of Applied Biobehavioral Research,**18*, 24–36. 10.1111/jabr.12005

[CR16] McGeary, D. D., Seech, T., Peterson, A. L., McGeary, C. A., Gatchel, R. J., & Vriend, C. (2012). Health care utilization after interdisciplinary chronic pain treatment: Part I. Description of utilization of costly health care interventions. *Journal of Applied Biobehavioral Research,**17*, 215–228. 10.1111/jabr.12001

[CR17] Mills, S., Torrance, N., & Smith, B. H. (2016). Identification and management of chronic pain in Primary Care: A review. *Current Psychiatry Reports,**18*, 22. 10.1007/s11920-015-0659-9 26820898 10.1007/s11920-015-0659-9 PMC4731442

[CR29] Noyek, S., Lund, T., Jordan, A., Hoppe, T., Mitchell, R., Mitchell, R., Stinson, J., & Noel, M. (2023). Exploring the Lived Experiences of Pain in Military Families: A Qualitative Examination. *The Journal of Pain*, *24*(12), 2340–2351. 10.1016/j.jpain.2023.07.016 10.1016/j.jpain.2023.07.016 37473902

[CR18] Office of the Army Surgeon General. (2010). *Pain Management Task Force: Final report*. U.S. Army Medical Department. http://www.dvcipm.org/site/assets/files/1070/pain-task-force-final-report-may-2010.pdf

[CR19] Ojala, T., Häkkinen, A., Karppinen, J., Sipilä, K., Suutama, T., & Piirainen, A. (2015). Although unseen, chronic pain is real–A phenomenological study. *Scandinavian Journal of Pain,**6*, 33–40. 10.1016/j.sjpain.2014.04.004 29911591 10.1016/j.sjpain.2014.04.004

[CR20] Pujol, L. A., Sussman, L., Clapp, J., Nilson, R., Gill, H., Boge, J., & Goff, B. (2015). Functional restoration for chronic pain patients in the military: Early results of the San Antonio Military Medical Center functional restoration program. US Army Medical Department Journal, 1–7. 26606402

[CR35] Polomano, R. C., Galloway, K. T., Kent, M. L., Brandon-Edwards, H., Kwon, K. N., Morales, C., & Buckenmaier, C., 3rd (2016). Psychometric testing of the Defense and Veterans Pain Rating Scale (DVPRS): A new pain scale for military population. *Pain Medicine (Malden, Mass.)*, *17*(8), 1505–1519.10.1093/pm/pnw105 10.1093/pm/pnw105 27272528

[CR28] Qureshi, A. R., Patel, M., Neumark, S., Wang, L., Couban, R. J., Sadeghirad, B., Bengizi, A., & Busse, J. W. (2025). Prevalence of chronic non-cancer pain among military veterans: a systematic review and meta-analysis of observational studies. *BMJ Military Health*, *171*(4), 310–314. 10.1136/military-2023-002554 10.1136/military-2023-002554 38124087

[CR21] Reed, D. E., Stump, T. E., Monohan, P. O., & Kroenke, K. (2024). Comparable minimally important differences and responsiveness of Brief Pain Inventory and PEG Pain Scales across 6 trials. *The Journal of Pain,**25*, 142–152. 37544394 10.1016/j.jpain.2023.07.028 PMC10859144

[CR22] Reiter, J. T., Dobmeyer, A. C., & Hunter, C. L. (2018). The Primary Care Behavioral Health (PCBH) model: An overview and operational definition. *Journal of Clinical Psychology in Medical Settings,**25*, 109–126. 10.1007/s10880-017-9531-x 29480434 10.1007/s10880-017-9531-x

[CR30] Roseen, E. J., Weinberg, J., & Saper, R. B. (2024). Yoga versus education for Veterans with chronic low back pain. *Journal of General Internal Medicine*, *39*(1), 141. 10.1007/s11606-023-08447-2 10.1007/s11606-023-08447-2 PMC10817863 37907773

[CR33] Robinson, P. J., & Reiter, J. T. (2025). Behavioral consultation and primary care: A guide to integrating services (3rd ed.). Springer Nature Switzerland AG.10.1007/978-3-031-72150-2

[CR23] Sherry, T. B., Roth, C. P., Bhandarkar, M., & Hepner, K. A. (2021). Chronic pain among service members: Using administrative data to strengthen research and quality improvement (Report No. RRA1160–1). RAND Corporation. https://www.rand.org/pubs/research_reports/RRA1160-1.html

[CR24] Stewart, M. O., Karlin, B. E., Murphy, J. L., Raffa, S. D., Miller, S. A., McKellar, J., & Kerns, R. D. (2015). National dissemination of cognitive-behavioral therapy for chronic pain in veterans: Therapist and patient-level outcomes. *The Clinical Journal of Pain,**31*, 722–729. 10.1097/AJP.0000000000000151 25171637 10.1097/AJP.0000000000000151

[CR32] Sandbrink, F., Murphy, J. L., Johansson, M., Olson, J. L., Edens, E., Clinton-Lont, J., Sall, J., Spevak, C., & VA/DoD Guideline Development Group (2023). The use of opioids in the management of chronic pain: Synopsis of the 2022 updated U.S. Department of Veterans Affairs and U.S. Department of Defense Clinical Practice Guideline. *Annals of internal medicine*, *176*(3), 388–397. 10.7326/M22-2917 10.7326/M22-2917 36780654

[CR25] Tai-Seale, M., Bolin, J., Bao, X., & Street, R. (2011). Management of chronic pain among older patients: Inside primary care in the US. *European Journal of Pain,**15*, 1087. e1081-1087. e1088. 10.1016/j.ejpain.2011.06.012 10.1016/j.ejpain.2011.06.012 PMC3208766 21784680

[CR27] Taylor, K. A., Kapos, F. P., Sharpe, J. A., Kosinski, A. S., Rhon, D. I., & Goode, A. P. (2024). Seventeen-Year National Pain Prevalence Trends Among U.S. Military Veterans. *The Journal of Pain*, *25*(5), 104420. 10.1016/j.jpain.2023.11.003 10.1016/j.jpain.2023.11.003 PMC11184511 37952861

[CR26] Toblin, R. L., Quartana, P. J., Riviere, L. A., Walper, K. C., & Hoge, C. W. (2014). Chronic pain and opioid use in US soldiers after combat deployment. *JAMA Internal Medicine,**174*, 1400–1401. 10.1001/jamainternmed.2014.2726 24978399 10.1001/jamainternmed.2014.2726

