# Supplementary material for: Targeting Chronic Pain in Primary Care Settings Using Behavioral Health Consultants: A Pilot Study Implementing Brief Cognitive Behavioral Therapy for Chronic Pain
Source: J Clin Psychol Med Settings. 2026 Jan 19;33(2):281–93. doi: 10.1007/s10880-025-10121-0 (PMC13226382; doi:10.1007/s10880-025-10121-0)
Supplement: Supplementary file 1 — Supplementary file1 (DOCX 16 KB) [file 10880_2025_10121_MOESM1_ESM.docx]

**Supplemental Table 1.** Standardized mean differences (SMD) between treatment completers and non-completers across key demographic and pain variables.

| Variable | Non-Completer | Completer | SMD |
| --- | --- | --- | --- |
| Gender = Female (%) | 5 (62.5) | 27 (77.1) | 0.323 |
| Age (mean (SD)) | 45.43 (13.61) | 44.66 (8.33) | 0.068 |
| Ethnicity = NonHispanic (%) | 7 (87.5) | 29 (82.9) | 0.131 |
| Race (%) |  |  | 0.708 |
| African American | 3 (37.5) | 11 (31.4) |  |
| Asian | 0 (0.0) | 1 (2.9) |  |
| Caucasian | 5 (62.5) | 17 (48.6) |  |
| Other | 0 (0.0) | 6 (17.1) |  |
| Marital (%) |  |  | 0.643 |
| Currently married | 8 (100.0) | 29 (82.9) |  |
| Currently separated or divorced | 0 (0.0) | 5 (14.3) |  |
| Not married but currently in a relationship and living with partner | 0 (0.0) | 1 (2.9) |  |
| Education (%) |  |  | 0.879 |
| 4-year College Degree | 4 (50.0) | 13 (37.1) |  |
| Associates Degree | 1 (12.5) | 5 (14.3) |  |
| High School Diploma | 1 (12.5) | 0 (0.0) |  |
| Master Degree | 0 (0.0) | 5 (14.3) |  |
| Some College | 2 (25.0) | 11 (31.4) |  |
| Some High School | 0 (0.0) | 1 (2.9) |  |
| DVPRS Current Pain (mean (SD))  DVPRS Average Pain (mean (SD)) | 5.12 (1.96)  5.62 (2.20) | 4.23 (2.24)  4.26 (2.76) | 0.426  0.548 |
